# Precision oncology for *RET*-related tumors

**DOI:** 10.3389/fonc.2022.992636

**Published:** 2022-08-24

**Authors:** Antonella Verrienti, Giorgio Grani, Marialuisa Sponziello, Valeria Pecce, Giuseppe Damante, Cosimo Durante, Diego Russo, Sebastiano Filetti

**Affiliations:** ^1^ Department of Translational and Precision Medicine, Sapienza University of Rome, Rome, Italy; ^2^ Department of Medicine, University of Udine, Udine, Italy; ^3^ Department of Health Sciences, University “Magna Graecia” of Catanzaro, Catanzaro, Italy; ^4^ School of Health, UNITELMA Sapienza University of Rome, Rome, Italy

**Keywords:** *RET* deletions, *RET* indels, acquired resistance, medullary thyroid cancer (MTC), *RET*-mutated cancers, pralsetinib, selpercatinib

## Abstract

Aberrant activation of the *RET* proto-oncogene is implicated in a plethora of cancers. *RET* gain-of-function point mutations are driver events in multiple endocrine neoplasia 2 (MEN2) syndrome and in sporadic medullary thyroid cancer, while *RET* rearrangements are driver events in several non-medullary thyroid cancers. Drugs able to inhibit RET have been used to treat *RET*-mutated cancers. Multikinase inhibitors were initially used, though they showed modest efficacy and significant toxicity. However, new RET selective inhibitors, such as selpercatinib and pralsetinib, have recently been tested and have shown good efficacy and tolerability, even if no direct comparison is yet available between multikinase and selective inhibitors. The advent of high-throughput technology has identified cancers with rare *RET* alterations beyond point mutations and fusions, including *RET* deletions, raising questions about whether these alterations have a functional effect and can be targeted by RET inhibitors. In this mini review, we focus on tumors with *RET* deletions, including deletions/insertions (indels), and their response to RET inhibitors.

## Introduction

**Figure 1:**
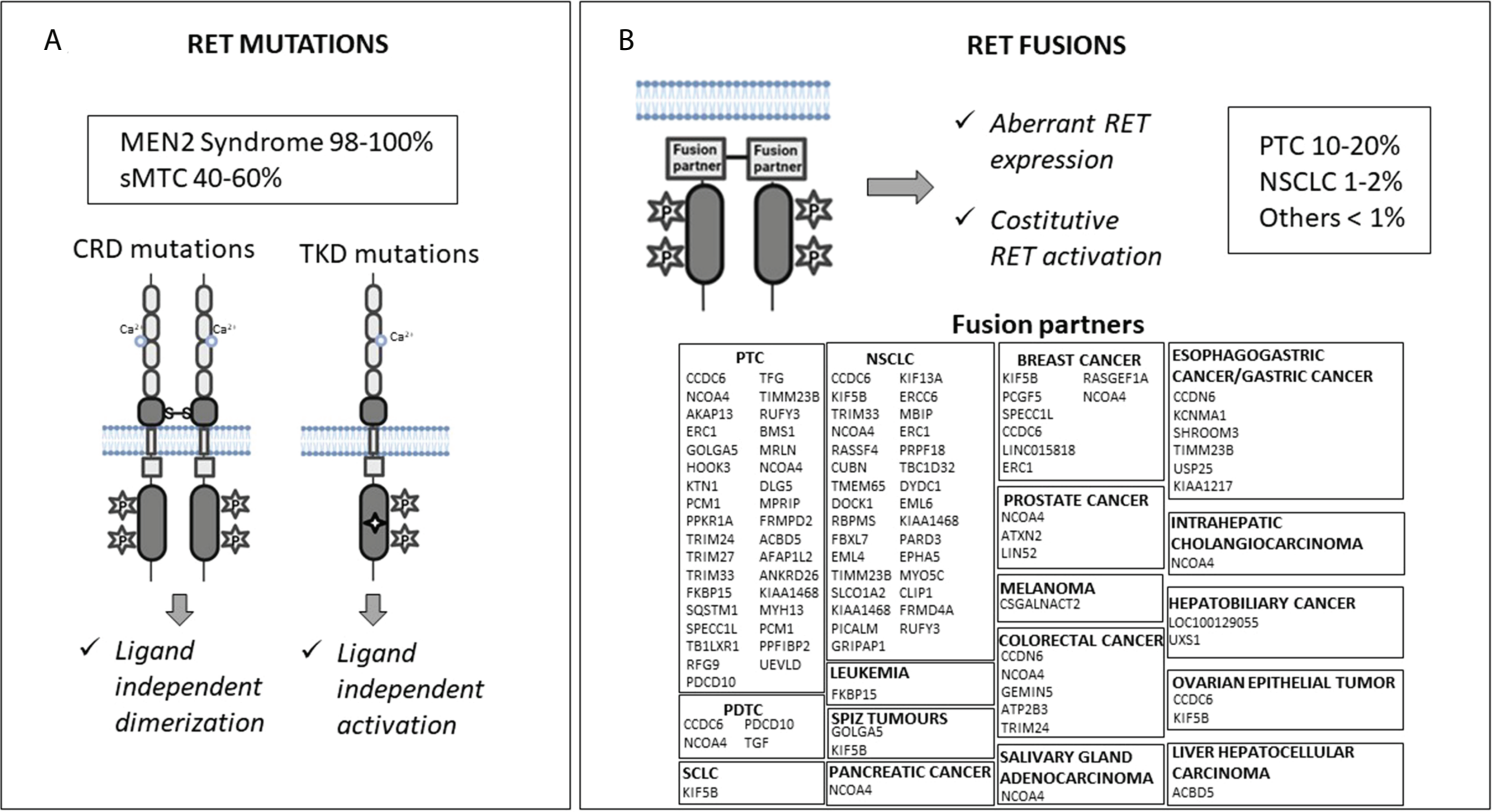
Molecular mechanisms of RET activation: mutations (Panel **A**) and fusions (Panel **B**). CRD, cysteine-rich domain; TKD, tyrosine kinase domain; sMTC, sporadic medullary thyroid cancer; PTC, papillary thyroid cancer; NSCLC, non-small cell lung cancer; PDTC, poorly differentiated thyroid cancer; SCLC, small cell lung cancer.

The *RET* proto-oncogene encodes for a transmembrane glycoprotein receptor with tyrosine kinase activity. It is involved in several cell processes during embryogenesis, including proliferation, differentiation, motility, and survival (1). *RET* gene mutations and fusions are known to be gain-of-function driver events in many cancer types (**Figure 1**).

*RET* germline gain-of-function mutations cause predisposition to multiple endocrine neoplasia 2 (MEN2) syndrome, while somatic *RET* mutations have been found in 40-65% of sporadic medullary thyroid cancers (MTCs) (2–5). *RET* mutations carry out their oncogenic effect through two mechanisms. Mutations located in the extracellular cysteine-rich domain can lead to RET receptor constitutive dimerization and activation regardless of the presence of ligands, while mutations in the tyrosine kinase domains can cause a conformational change in the intracellular tyrosine kinase binding pocket, which allows constitutive kinase activation and altered substrate binding (1, 6–9) (**Figure 1A**).

Sanger sequencing is preferentially used for the detection of germline mutations in MEN2 syndrome; however, in evaluating the presence of somatic mutations, it may generate false negative results since it is not able to detect mutations below 15-20% of allele frequency. Alternatively, quantitative polymerase chain reaction (qPCR) or digital PCR can be used for the screening of somatic hotspot mutations, reaching a limit of detection of around 1% of allelic frequency or, in the case of digital PCR, less than 1% (10). The advent of high-throughput technologies, such as next-generation sequencing (NGS), has allowed hundreds to thousands of genes to be simultaneously analyzed, thus increasing the detection of novel or rare variants with a high sensitivity.

*RET* rearrangements occur in 10–20% of papillary thyroid carcinomas (PTCs), 1–2% of non-small cell lung cancers (NSCLCs), and in <1% of other cancers (e.g., colorectal cancer, breast cancer, chronic myelomonocytic leukemia, ovarian and salivary gland cancers, etc.) (11). *RET* rearrangements produce chimeras formed by the juxtaposition of an N-terminal partner to the *RET* C-terminal portion, including its catalytic domain. This leads to aberrant *RET* overexpression, ligand-independent dimerization, and kinase activation (**Figure 1** **B**). Notably, the RET fusion partner may influence the oncogenic potential of the chimeric RET protein affecting the intracellular location of the RET kinase (consequently the activated signaling pathways), and its expression levels (12). In addition, the altered function of the fusion partner may also have a role in the neoplastic transformation (13, 14).

Fluorescent *in situ* hybridization (FISH) is considered the standard used for the detection of *RET* rearrangements and has good sensitivity and specificity (10, 15). However, it may not be adequately informative regarding the specific RET fusion unless a specific fusion partner probe can be used (10, 15). Moreover, FISH results are difficult to interpret in many circumstances, such as in presence of pericentric fusions, deletions, and when possible partner genes are in close proximity to the *RET* gene (10). Reverse transcription-polymerase chain reaction (RT-PCR) can identify specific known *RET* fusion partners, but since it uses preselected primers, it is not able to detect novel fusion partners. Thus, it could underestimate the presence of *RET* rearrangements. Since this methodology is not ideal for the degraded and poor RNA quality isolated from formalin-fixed paraffin-embedded tissues, it is generally used together with other methodologies, such as immunohistochemistry (IHC) and FISH (10, 15). IHC is currently not indicated for the screening of *RET* alterations due to the high false positive and negative rates (15). Although DNA-based NGS can be designed for gene fusion detection, it doesn’t achieve high sensitivity and an RNA-based NGS is preferred (10).

The comprehensive genetic profiling of tumors made possible by novel detection technologies has resulted in the identification of multiple cancers with rare *RET* alterations beyond point mutations and fusions (16, 17), including a nonnegligible number of *RET* deletions. In this review, *RET* deletions also include deletions/insertions (indels).

## *Ret* deletions in cancers

*RET* deletions are not frequently found in MTC. They have been reported in 5% of all *RET*-mutated sporadic MTCs, as reported in the Catalogue of Somatic Mutations in Cancer (COSMIC database: https://cancer.sanger.ac.uk/cosmic, accessed June 2022), and they represent around 3.5% of all germlines *RET* alterations found in MEN2 patients, as reported in the ARUP database (https://arup.utah.edu/database/MEN2/MEN2_display.php accessed June 2022). In non-MTC cancers, their frequency is very low, ranging from 0.03% (COSMIC database) to 0.2% (cBioPortal for Cancer Genomics public databases; https://www.cbioportal.org/, accessed June 2022).

### *Ret* in-frame deletions

Overall, 37 *RET* deletions have been described in MTC patients and almost all of them (36/37, 97%) are in-frame. Seven are germline deletions and are mainly located in the cysteine-rich domain, at exons 11 and 10 (18–24), and two are in the cadherin-like coding regions, at exons 6 and 7 (25, 26). Most *RET* deletions have been found in the tumor tissue of sporadic MTCs, mainly at exons 11 and 15 (3, 4, 25, 27–49), and to a lesser extent at exons 10 and 8 (30, 45, 49–51). Although only a few deletions have been reported in non-”hotspot” exons (i.e., exons 6 and 7), we cannot exclude that their frequency may be higher since those exons are not routinely studied. The prognostic role of the RET deletions (including indels) has not been clearly proved due to the few available data. However, in a recent paper, Elisei R. et al. observed that MTC harboring RET indels, show a more aggressive phenotype with a high prevalence of advanced cases at diagnosis (45).

*RET* in-frame deletions have also been described in other cancer types, as reported in the COSMIC and cBioPortal databases (52, 53). Fifteen in-frame *RET* deletions have been found in 30 oncologic patients. Interestingly, 6/30 patients (20%) are affected by pheochromocytoma (PHEO) and carry deletions in common with MTCs, mapping at *RET* exons 11 and 15. This is not surprising since both MTC and PHEO can be induced by activating *RET* alterations. The remaining in-frame *RET* deletions have been observed in breast, large intestine, gastric, pancreatic, kidney, and lung cancers.

### *Ret* frameshift deletions

Only one *RET* frameshift deletion, p. Gln681Argfs*50, has been reported in an MTC patient. However, it was found in copresence with the RET p.A680T point mutation and its functional effect has not yet been demonstrated (36). Conversely, a greater number of *RET* frameshift deletions has been described in other cancers, as reported in COSMIC and cBioPortal public databases (52, 53). These deletions are spread out along the gene, including the hotspot exons.

Frameshift deletions are commonly loss-of-function alterations since they result in a shift of the reading frame used for protein translation, leading to a completely different sequence of the polypeptide. They often introduce an early stop codon resulting in a truncated protein. However, the major mechanism explaining the loss of function is nonsense-mediated mRNA decay, by which mutated mRNA is degraded (54).

It has yet to be proven whether RET in-frame and frameshift deletions in non-MTC and non-PHEO cancers are pathogenic.

## *Ret*-targeted therapies

The identification of key driver oncogenes as targetable activated kinases has allowed clinicians to explore new treatment options. Therefore, multikinase inhibitors (MKIs) that target multiple tyrosine kinase receptors, including RET and those involved in angiogenesis, such as VEGFRs and PDGFRs, were initially used to treat advanced *RET*-mutated MTC and subsequently other *RET*-altered cancers (55, 56). Given their multi-target inhibition, it is not clear whether their observed antitumor activity is due to RET inhibition or the inhibition of other kinase targets (57, 58) (**Table 1**).

**Table 1:** Drugs targeting medullary thyroid cancers and RET-mutated NSCLC with relevant clinical trial data.

MTC-targeting agents	IC50 (nM) for RET (11)	Targets	Study phase	Mutations	ORR	mPFS*	mOS*	NCT
**Multitarget kinase inhibitors**								
Vandetanib	0.13	VEGFR2-3, EGFR, RET	III	*RET*+*RAS*+unknown	45	30.5	NR	NCT00410761
Cabozantinib	5.2	VEGFR2, KIT, FLT-3, RET, MET	III	*RET+RAS*+ unknown	28	11.2	26.6	NCT00704730
				M918T negative	20	20.2	5.7	
				M918T	34	13.9	44.3	
Sorafenib	5.9	BRAF, KIT, FLT-3, VEGFR2, PDGFR	II	Not assessed	25	NR	NR	NCT02114658
Lenvatinib	1.5	VEGFR1-3, FGFR1-4, PDGFRa, KIT, RET	II	*RET+RAS*	36	9	16.6	NCT00784303
Anlotinib		VEGFR1-3, FGFR1-4, KIT FGFR	II	Not assessed	48.4	22.4	50.4	NCT02586350
Sunitinib	5	PDGFR, KIT VEGFR1-3, FLT-3, RET	II	Not assessed	38.5	16.5	29.4	NCT00510640
*Investigational*								
Regorafenib	1.5	BRAF, VAGFR1-3 PDGFRa/b, RET, KIT, FGFR1-2	II	-	-	-	-	NCT02657551
**Selective RET-targeting inhibitors **								
Pralsetinib	0.4	RET, VEGFR2	I/II	*RET*/previous TKI	60	NR	NR	NCT03037385
				*RET*/TKI naïve	71	NR	NR	
Selpercatinib	0.4	RET, VEGFR2	I/II	*RET*/Previous TKI	69	NR (1-year PFS 82%)	NR	NCT03157128
				*RET*/TKI Naïve	73	NR (1-year PFS 92%)	NR	
*Investigational*								
TPX-0046		RET	I/II	*RET* alterations	-	-	-	NCT04161391
TAS0953/HM06		RET	I/II	*RET* alterations	–	–	–	NCT04683250
BOS172738		RET	I	*RET* alterations	-	-	-	NCT03780517
SL-1001#		RET	–	–	–	–	–	–
***RET*-mutated NSLC-targeting agents**								
Selpercatinib (first line)	0.4	RET, VEGFR2	I/II	RET fusion-positive	85	NR	−	NCT03157128
Selpercatinib (previously received at least platinum-based chemotherapy)			I/II	RET fusion-positive	64	16.5	−	NCT03157128
Selpercatinib			II	RET fusion-positive	−	−	−	NCT04268550
Selpercatinib vs. carboplatin/cisplatin + pemetrexed ± pembrolizumab			III	RET fusion-positive	−	−	−	NCT04194944
Pralsetinib (first line)	0.4	RET, VEGFR2	I/II	RET fusion-positive	70	9.1	NR	NCT03037385
Pralsetinib (previously received platinum-based chemotherapy)			I/II	RET fusion-positive	61	17.1	NR	NCT03037385
Pralsetinib vs. carboplatin/cisplatin + pemetrexed ± pembrolizumab or carboplatin/cisplatin Gemcitabine (squamous histology)			III	RET fusion-positive	−	−	−	NCT04222972
Vandetanib	0.13	VEGFR2-3, EGFR, RET	II	RET fusion-positive	18	4.5	11.6	NCT01823068
Cabozantinib	5.2	VEGFR2, KIT, FLT-3, RET, MET	II	RET, ROS1, NTRK fusions or increased MET or AXL activity	28	5.5	9.9	NCT01639508
			II	RET fusion-positive	−	−	−	NCT04131543
CPI (nivolumab or pembrolizumab)	-	-	RS#	at least one oncogenic driver alteration (*KRAS, EGFR, BRAF, MET, HER2, ALK, RET, ROS1*)	25	2.1	21.3	(59)#
Platinum-pemetrexed (first line)	-	-	RS#	RET fusion-positive	50	9.2	26.4	(60)#
TAS0953/HM06		RET	I/II	*RET* alterations	−	−	−	NCT04683250
BOS172738		RET	I	*RET* alterations	−	−	−	NCT03780517
TPX-0046		RET	I/II	*RET* alterations	−	−	−	NCT04161391

ORR, objective response rate; OS, overall survival; PFS, progression-free survival; NR, not reached; RS, retrospective study; *months; # clinical trials still not available. Adapted from (58) and (61).

Cabozantinib and vandetanib have been approved for first-line treatment in MTC regardless of *RET* mutational status, even if the presence of *RET* mutations, particularly the *RET* p.M918T mutation, seems to be associated with a better response to cabozantinib in terms of overall response rate and progression-free survival (62, 63). Similarly, M918T mutation-positive patients also showed a higher response to vandetanib (64). Vandetanib showed a higher median progression-free survival (mPFS) than placebo (30.5 vs 19.3 months) in the ZETA trial (64), as had cabozantinib in the EXAM trial (11.2 vs 4.0 months) (65). The clinical effectiveness of vandetanib and cabozantinib in advanced MTC patients was also confirmed from real-world data, showing a mPFS up to 47 months for vandetanib (66–68) and up to 4 months for cabozantinib (66). The median overall survival (OS) for vandetanib and cabozantinib was 53 months and 24 months, respectively, in the German real-world multicenter cohort (66).

MKI treatment of *RET-*rearranged NSCLC showed a modest clinical benefit that was lower than that observed with EGFR, ALK, and ROS1 inhibitors (61) (**Table 1**). Moreover, MKI response can differ depending on the fusion partner. For example, vandetanib showed a greater effect in *CCDC6-RET* fusion tumors compared with *KIF5B-RET* (57). However, the adverse effects of non-selective RET inhibitors observed in all treated tumors due to their off-target side effects are responsible for high discontinuation and dose reduction rates (e.g., 12% and 35% for vandetanib and 16% and 79% for cabozantinib when used as thyroid cancer treatments, respectively) (57).

In the last years, small and highly selective RET inhibitors have been designed to overcome the treatment-related toxicities of non-selective RET inhibitors and acquired resistance to them (57). The new selective RET inhibitors pralsetinib (LOXO-292) and selpercatinib (BLU-667) have demonstrated both good efficacy and tolerability: in phase I/II trials, the mPFS was not reached, and the overall response rate was 71% and 73% (first line treatment), and 60% and 69% (second line treatment), respectively (**Table 1**). Currently, both drugs have been approved by the Food and Drug Administration (FDA) for the treatment of patients more than 12 years of age with: i) advanced or metastatic *RET*-mutant MTC; ii) RET fusion-positive metastatic NSCLC, and iii) advanced or metastatic RET fusion-positive thyroid cancer patients who require systemic therapy and who are radioactive iodine refractory. These drugs also show robust activity in other *RET* alteration-positive solid tumors (69). Interestingly, both drugs seem to be effective regardless of previous MKI or immune checkpoint therapies (57). Selpercatinib is also reported to be effective on CNS metastases (70) and uncommon metastatic sites, such as choroidal metastases (71). Common side effects of selpercatinib include dry mouth, hypertension, fatigue, increased aspartate aminotransferase level (AST), increased alanine aminotransferase level (ALT), increased glucose levels, and hypocalcemia, while pralsetinib additionally caused pain, constipation, and hematological toxicities such as decreased lymphocytes, neutrophils, and hemoglobin. Interstitial pneumonia is also reported for pralsetinib. The discontinuation and dose reduction rates in phase I/II trials were 2% and 30% for selpercatinib (72), and 4% and 44% for pralsetinib (73).

Given the availability of these drugs, the screening for and detection of *RET* driver alterations is now crucial in clinical practice since it provides more targeted treatment options. Unlike multitarget inhibitors, pralsetinib and selpercatinib have a selective nanomolar potency against RET and a diverse set of *RET* fusions and mutations. Head-to-head studies directly comparing efficacy and safety of selective RET inhibitors with MKI are currently ongoing (NCT04760288, NCT04211337); results are not yet available.

### Specific mutations and acquired resistance

Some specific mutations are expected to cause acquired resistance to MKI treatments (12). Preclinical studies have shown that acquired gatekeeper mutation V804L is associated with MKI resistance (12). The emergence of a V804M mutation was reported in a patient with RET-mutant, sporadic MTC treated previously with multiple MKIs (74). Emergent V804L and S904F mutations were reported in patients with RET fusion-positive NSCLC during treatment with vandetanib (75, 76). The frequency, prognostic role, and clinical actionability of these mutations are not entirely clear (75). Some preclinical models identified other resistance mutations, including the V804E, G810A/S/R, I788N, 730I, E732K, V871I, V738A, A807V, F998V and Y806N (13, 77, 78).

Selpercatinib has a specific binding modality: both front and back pockets of RET are occupied without being affected by V804 mutations (unlike other tyrosine kinase inhibitors) (11). Selpercatinib was developed to be effective in RET^V804L^ and RET^V804M^ gatekeeper mutations and was found to be 60–1300 fold more effective than multitarget inhibitors against cell lines engineered with KIF5B-RET^V804L/M^ gatekeeper mutations (74).

Conversely, *RET* mutations at the C-lobe solvent front (RET p.G810C/S/R), hinge (RETY p.806C/N), and β2 strand (RET p.V738A, only identified in cell lines) cause acquired resistance to selpercatinib (61, 79–81). Structural modeling showed that selpercatinib binding to the kinase ATP/selpercatinib binding site can be hindered if the glycine residue at position 810 in the RET solvent front is substituted with charged or polar residues (79, 80). *In vitro* experiments using BaF3/KIF5B-RET cells showed that pralsetinib and selpercatinib bind to RET in a similar mode and both are resistant to the same mutations (80), although some mutations (i.e., L730V/I) seem to be resistant only to pralsetinib (82).

### New selective inhibitors in clinical development

New selective RET inhibitors are under development (**Table 1**). TPX-0046 is a dual RET/SRC kinase inhibitor, with activity in drug-resistant and naïve *RET*-driven cancer models. It is in phase I/II clinical trials for advanced solid tumors harboring *RET* fusions or mutations (NCT04161391). TAS0953 (HM06) is undergoing a phase I/II study in patients with advanced solid tumors with *RET* gene abnormalities (NCT04683250). SYHA1815 has an approximately 20-fold selectivity for RET over VEGFR2 and is being studied in a phase I trial in China (83). Other potential drug compounds, such as LOX-18228, LOX-19260, BOS172738 (DS-5010), and SL-1001 (84–86), are still in the preclinical stage. There are also research efforts to obtain mutant-selective inhibitors that may offer clinical advantages.

### Clinical response in patients with ret deletions

Despite efforts to develop super-selective inhibitors, data available on the response of cancers harboring *RET* deletions to selective RET inhibitors are scarce and concern only MTCs. A *RET* p.D378_G385delinsE MTC was treated with selpercatinib and achieved partial response, with a maximum tumor reduction of 86% (87). The treatment of two *RET* p.L629_D631delinsH MTCs, one with cabozantinib and the other with a combination of sorafenib and tipifarnib, showed a partial response, with a tumor reduction of 48% and 46%, respectively (43, 44). Two MTCs with *RET* p.E632_636del and p.L633_A639del were treated with vandetanib and cabozantinib, respectively, showing stable disease (25, 43). In one case, disease progression was observed after seven months of treatment (22). Recently, two MTC patients with the p.E632_L633del and p.D631_L633delinsS *RET* deletion, respectively, who were previously treated with cabozantinib and/or vandetanib, experienced a treatment benefit with selpercatinib, with a rapid biochemical response. In particular, the first patient showed a partial response in the target lesions and stable disease in non-target lesions, while the second patient showed stable disease and a partial response in target and non-target lesions, respectively (45). An *in vitro* study provided evidence that the p.C630del *RET* alteration is sensitive to pralsetinib (23). A *RET* p.D898_E901del MTC was treated with cabozantinib, showing stable disease (43). Lastly, Zhao et al. used mutant-transformed Ba/F3 cells to demonstrate that p.D898_E901del is sensitive to selpercatinib and pralsetinib (88).

## Discussion

The advancement of sequencing technologies has allowed comprehensive genetic profiling of tumors and the identification of new *RET* alterations, including deletions. Although the reported tumors with *RET* deletions are few, we cannot exclude that their real prevalence may be higher. Indeed, in clinical practice, *RET* deletions are usually not investigated through the gene.

Data on *RET* deletions as driver alterations in cancer are still scarce. In MTC and PHEO, only *RET* in-frame deletions have been reported, supporting their possible gain-of-function role. For a few of them, their oncogenic potential has been demonstrated through *in vitro* experiments (21, 23, 26, 49, 88–90). Conversely, frameshift deletions have been observed in a wide range of tumor types, except MTC and PHEO, though their functional role as driver alterations has not yet been demonstrated.

To date, limited information about the response of tumors with *RET* deletions to RET inhibitors is available and only concerns MTC patients. In those patients, treatment efficacy seems to be comparable to MTCs with *RET* point mutations. Considering the potential benefit of treating tumors with RET inhibitors, it is crucial to understand the real impact of these deletions in cancer development and progression and their response to *RET* targeted therapies.

## Author contributions

Conceptualization, SF and CD. Writing—original draft preparation AV and GG. Writing—review and editing, MS, VP, DR, and GD. Supervision, SF and CD. All authors have read and agreed to the published version of the manuscript.

## Acknowledgments

The manuscript was edited by Melissa Kerr.

## Conflict of interest

The authors declare that the research was conducted in the absence of any commercial or financial relationships that could be construed as a potential conflict of interest.

## Publisher’s note

All claims expressed in this article are solely those of the authors and do not necessarily represent those of their affiliated organizations, or those of the publisher, the editors and the reviewers. Any product that may be evaluated in this article, or claim that may be made by its manufacturer, is not guaranteed or endorsed by the publisher.
